# Successful ablation of atrial fibrillation does not normalise left ventricular function, reverse impaired myocardial energetics or increase perfusion reserve: novel mechanistic insights with clinical implications

**DOI:** 10.1186/1532-429X-18-S1-O35

**Published:** 2016-01-27

**Authors:** Rohan S Wijesurendra, Alexander Liu, Christian Eichhorn, Eylem Levelt, Rina Ariga, Yaver Bashir, Matthew Ginks, Kim Rajappan, Timothy R Betts, Vanessa M Ferreira, Barbara Casadei, Stefan Neubauer

**Affiliations:** 1 grid.4991.50000000419368948University of Oxford, Oxford, UK; 2 grid.410556.30000000104401440Oxford University Hospitals NHS Trust, Oxford, UK

## Background

Atrial fibrillation (AF) is associated with increased risk of heart failure and premature death, and with resistance to treatment. Animal models of pacing-induced AF indicate that AF-induced endothelial dysfunction, impaired coronary reserve, and myocardial remodelling are important in arrhythmia maintenance; however, human AF may reflect a subclinical cardiomyopathy that develops with ageing and risk factors, persists after restoration of sinus rhythm (SR), and provides a substrate for AF recurrence. To test this hypothesis, we investigated the effect of restoring SR by catheter ablation on left ventricular (LV) function, perfusion and energetics.

## Methods

72 subjects were recruited: 52 patients (63 ± 8 y) referred for AF ablation, and 20 age-matched controls (62 ± 7 y) in SR. Patients had symptomatic paroxysmal (n = 27) or persistent (n = 25) AF without coronary artery disease, valve disease, diabetes, uncontrolled hypertension, inflammatory disease or inadequate ventricular rate-control. CMR-derived short axis cines were analysed by an investigator blinded to rhythm and clinical status to calculate LV volumes and ejection fraction (EF). ^31^Phosphorus MR spectroscopy determined LV energetics (ratio of phosphocreatine to ATP - PCr/ATP), and adenosine stress/rest CMR assessed first-pass perfusion. Ablation success was evaluated by prolonged intermittent ECG monitoring, after a 3-month blanking period. A majority of patients (n = 30) were re-assessed 7 ± 1 months post-ablation.

## Results

**Figure 1:**
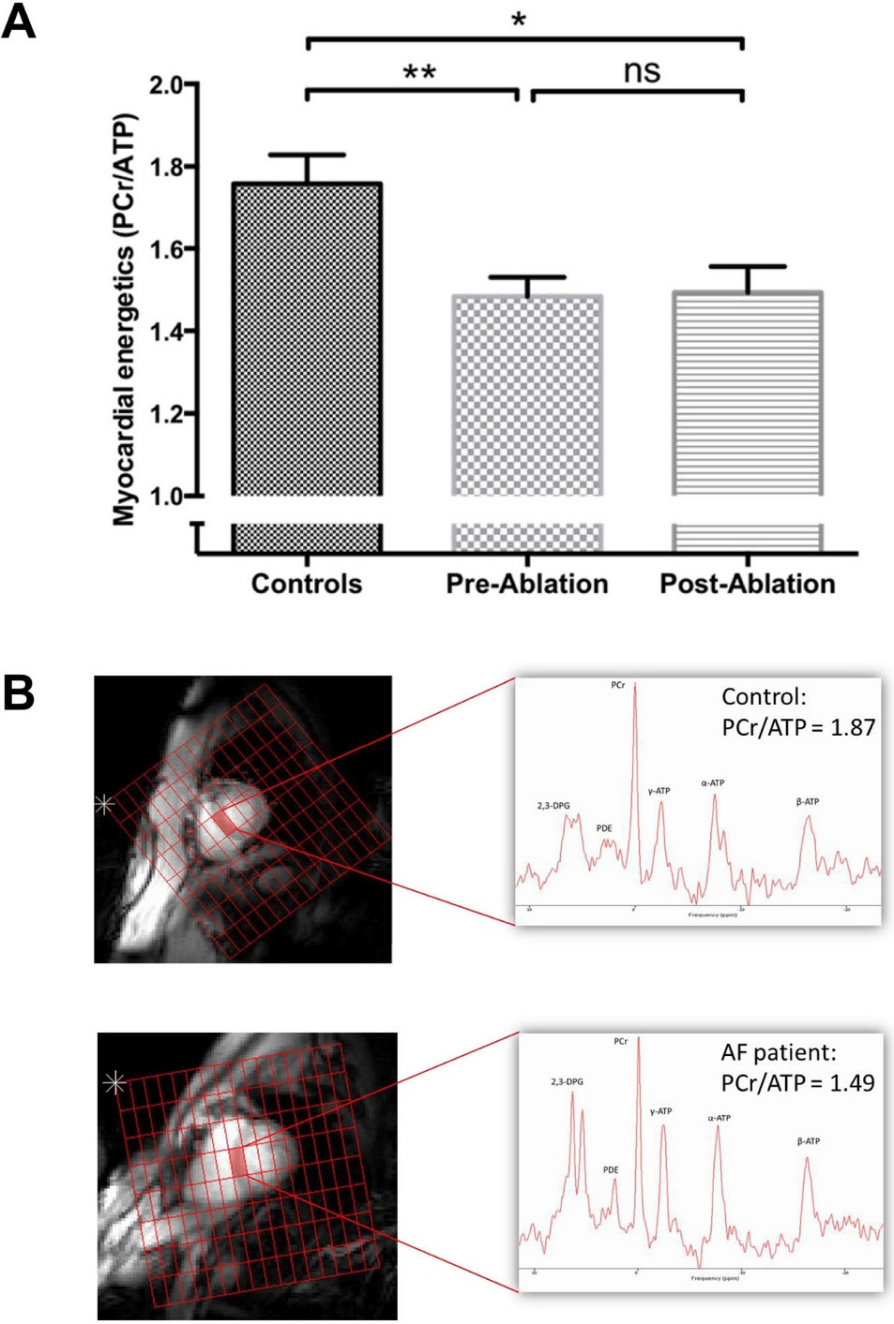
**Myocardial energetics is impaired in patients compared to controls, and does not improve post-ablation (** ***A*** **)**. Representative 31P spectra from a mid-ventricular septal voxel in a control (PCr/ATP ratio 1.87) and an AF patient (PCr/ATP ratio 1.49) are shown in (*B*). PCr/ATP = ratio of phosphocreatine to ATP. One-way ANOVA with post-hoc Bonferroni correction; * indicates p < 0.05, ** indicates p < 0.01; error bars indicate SEM.

In patients compared to controls, LVEF and energetics (Figure 1) were both significantly impaired (59 ± 10 vs 69 ± 6%, p < 0.001, and PCr/ATP 1.48 ± 0.33 vs 1.76 ± 0.31, p = 0.001, respectively). In patients, presence of AF rather than SR at the pre-ablation scan was associated with reduced LVEF (54 ± 9 vs 66 ± 7%, p = 0.001) but not energetics (PCr/ATP 1.49 ± 0.31 vs 1.59 ± 0.39, p = 0.50).

**Figure 2:**
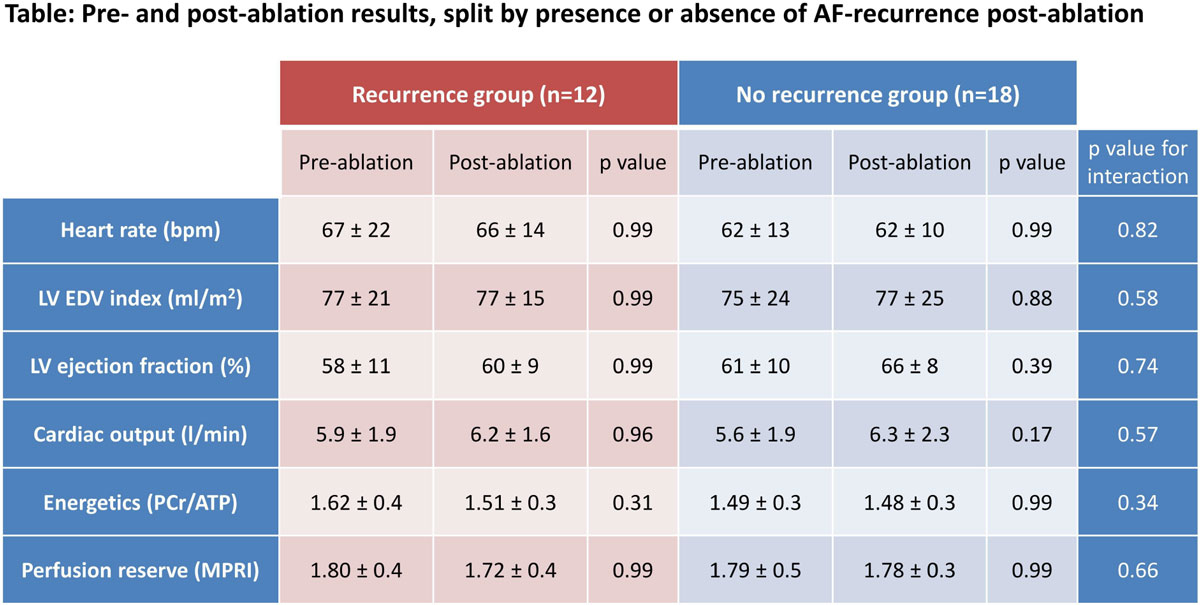
**Values are mean ± standard deviation**. **AF** indicates atrial fibrillation; **LV**, left ventricular; **EDV**, end-diastolic volume; **PCr/ATP**, ratio of phosphocreatine to adenosine triphosphate; **MPRI**, myocardial perfusion reserve index. Each parameter analysed using a 2-way repeated measures ANOVA - post-hoc Bonferroni-corrected p values are shown for pre/post ablation change in each group; p values are also shown for interaction between ablation success (i.e. recurrence or no recurrence of AF) and change in parameter post-ablation.

In patients with a rhythm of AF during pre-ablation CMR and SR during post-ablation CMR (n = 10), there was modest improvement, but not normalisation, in LVEF (63 ± 7 post-ablation vs 55 ± 9% pre-ablation, p = 0.02; post-ablation/control comparison p = 0.02). However, energetics and perfusion reserve were unchanged despite recovery of SR (both p = ns). Furthermore, when patients were grouped based on presence/absence of recurrent AF on ECG monitoring post-ablation, LV function, energetics and perfusion reserve were all unchanged, irrespective of ablation success (all p = ns; Figure 2).

## Conclusions

Even "lone" AF is associated with LV dysfunction - this is only partially explained by haemodynamic effects of AF at the time of assessment, as LV function does not normalise in patients who recover SR post-ablation. Moreover, successful ablation fails to reverse energetic impairment or increase perfusion reserve. These novel findings suggest that human AF may be the consequence (rather than the cause) of an occult cardiomyopathic process. Comprehensive therapeutic strategies that target and reverse this phenotype may reduce AF recurrence and improve clinical outcomes.

